# Impaired Coronary Microcirculation and Myocardial Systolic Function: A Narrative Review on Non-Invasive Assessment in Cardiovascular Diseases

**DOI:** 10.3390/life15091350

**Published:** 2025-08-26

**Authors:** Evangelos Tatsis, Constantinos Papadopoulos, Dimitrios Oikonomidis, Lampros Lakkas, Katerina K. Naka

**Affiliations:** 1 Second Department of Cardiology, “Korgialenio Mpenakio”—Hellenic Red Cross Hospital, 11526 Athens, Greece; papcost@gmail.com (C.P.); dimitris_oikon@yahoo.gr (D.O.); 2 Department of Physiology, Faculty of Medicine, School of Health Sciences, University of Ioannina, 45500 Ioannina, Greece; l.lakkas@uoi.gr; 3 Second Department of Cardiology, Faculty of Medicine, School of Health Sciences, University of Ioannina, 45500 Ioannina, Greece; anaka@uoi.gr

**Keywords:** CMD, CFR, coronary microcirculation, systolic dysfunction, non-invasive assessment

## Abstract

Coronary microvascular dysfunction (CMD) is presently recognized as a condition characterized by a reduction in coronary blood flow. CMD is associated with poor cardiac outcomes, and mounting evidence suggests that coronary microcirculation may be impaired in various cardiology pathologies. Non-invasive assessment of CMD remains challenging, and several imaging modalities—positron emission tomography (PET), computed tomography (CT), cardiac magnetic resonance (CMR), myocardial contrast echocardiography (MCE), and transthoracic Doppler echocardiography (TTE)—are proven to quantify coronary flow reserve (CFR) and myocardial blood flow (MBF), both valuable markers of CMD. However, each modality is limited by availability, standardization, and diagnostic utility. Furthermore, a growing number of studies have attempted to correlate CMD indices with systolic myocardial function markers, such as left ventricular ejection fraction, global longitudinal strain, and myocardial work indices. This review refers to the current evidence on CMD imaging and examines the association between CMD indices and systolic function in cardiovascular diseases. Understanding the impact of CMD on myocardial function is essential, as it highlights the coronary microcirculation impairment pathway as a potential target for therapeutic strategies.

## 1. Introduction

Coronary microcirculation is a vascular system that consists of pre-arterioles (diameter < 500 μm), arterioles (<200 μm), and capillaries, responsible for supplying sufficient perfusion to the myocardium, based on oxygen and metabolic demands. This adaptive complex system relies on the interaction of vascular and parenchymal cells and ensures optimal blood flow. As a result, in situations where oxygen demands are increased, the vascular tone is decreased to maintain coronary blood flow, a process largely regulated at the level of the coronary microcirculation, which accounts for approximately 90% of the total coronary vascular resistance.

Coronary microvascular dysfunction (CMD) is presently recognized as a condition resulting from a multifactorial interplay of molecular and structural processes, in which oxidative stress and inflammation play a leading role [1]. The pivotal mechanism involves the overproduction of reactive oxygen species (ROS), particularly by NADPH oxidase (Nox) enzymes and mitochondrial dysfunction. This biochemical imbalance lowers nitric oxide (NO) bioavailability by enhancing its conversion to peroxynitrite, which further inhibits endothelial nitric oxide synthase (eNOS) activity. The resulting endothelial dysfunction decreases vasodilatory responses and enhances vasoconstriction by increased endothelin-1 activity and RhoA/Rho-kinase pathway activation [2]. In addition to NOx enzymes and mitochondrial dysfunction, various other enzymatic and non-enzymatic sources contribute to ROS production in the coronary microcirculation. Xanthine oxidase, upregulated during ischemia–reperfusion, generates superoxide and hydrogen peroxide, intensifying oxidative stress in microvascular endothelial cells [3]. Uncoupled eNOS, often due to tetrahydrobiopterin (BH_4_) deficiency or oxidative degradation, shifts from producing NO to superoxide, further depleting NO availability. Cyclooxygenases (particularly COX-2) [4] and lipoxygenases generate ROS in inflammatory conditions. Additionally, myeloperoxidase, released from activated neutrophils and monocytes, produces oxidants, like hypochlorous acid, that damage microvascular endothelium. Peroxisomal β-oxidation contributes further by producing hydrogen peroxide [5]. Together, these alternative ROS sources amplify oxidative stress, worsen endothelial dysfunction, increase vasoconstriction, and play a critical role in the development of CMD.

In this narrative review, the authors aim to summarize current evidence on non-invasive imaging modalities developed to assess coronary microvascular dysfunction and to examine the relationship between CMD indices and left ventricular systolic function across a spectrum of cardiovascular diseases. The studies included in this review were retrieved from PubMed, and findings are reported according to GRADE certainty levels [6].

## 2. Coronary Microvascular Dysfunction

Functionally, CMD can be expressed as blunted microvascular relaxation or increased vasoconstrictive activity via both endothelium-dependent and -independent mechanisms. Structurally, CMD is marked by small coronary vessel remodeling such as medial thickening, perivascular fibrosis, and capillary rarefaction, especially in left ventricular hypertrophy or cardiomyopathies. The above pathophysiologic mechanisms underline a reduction in coronary blood flow and emphasize the multifactorial etiology of CMD [7].

Invasive and non-invasive methods can be used for the functional assessment of coronary microcirculation.

### 2.1. Invasive Assessment

Coronary angiography constitutes a comprehensive technique with the potential to visualize the anatomy of epicardial arteries, exclude significant coronary artery disease (CAD), and assess the microvascular function simultaneously. Indices particularly of value are the Coronary Flow Reserve (CFR), the Index of Microvascular Resistance (IMR), and the Hyperemic Microvascular Resistance (HMR). CFR is characterized as the ratio of hyperemic to the basal coronary flow velocity, and it reflects the vasodilatory capacity of the coronary microcirculation. It is predominantly assessed either with intracoronary Doppler or by coronary thermodilution approaches. CFR values > 2.5 are within normal, while values < 2.0 imply microvascular disease. Additionally, intracoronary CFR cannot always distinguish between diffuse atherosclerosis and true microvascular dysfunction. Its clinical interpretation can be complex, especially in patients with microvascular angina without obstructive coronary artery disease; in such circumstances, evaluation should include simultaneous measurement of fractional flow reserve (FFR) of epicardial stenosis. IMR, as the product of distal coronary pressure and saline mean transit time in hyperemia, represents a more hemodynamic and CAD-independent evaluation of microvascular dysfunction. HMR, from distal coronary pressure and maximum flow velocity determined by Doppler, yields another index with less variability than CFR but lacks widespread standardization and normative cut-off values, limiting clinical applicability. Dual-sensor guidewires enable simultaneous pressure and flow measurement, which can be utilized to calculate hyperemic stenosis resistance (HSR) and more clearly differentiate epicardial and microvascular disease, but their reliance on high-quality Doppler signals can lead to variability and limited reproducibility in clinical settings. Absolute coronary blood flow (CBF) and coronary blood flow velocity are other invasive parameters that further characterize coronary hemodynamics [8].

### 2.2. Non-Invasive Assessment (Table 1)

#### 2.2.1. Cardiac Positron Emission Tomography (PET)

Cardiac PET is the non-invasive gold standard method for the measurement of myocardial blood flow (MBF), coronary flow reserve (CFR), and for acquiring information regarding CMD. Using well-established tracers labeled with isotope-emitting positrons, such as 13N-ammonia and 82 rubidium, and pharmacologic vasodilators such as adenosine or dipyridamole, PET accurately measures MBF at rest and maximal hyperemia. These quantitative tests extend beyond the detection of obstructive CAD to detect early vascular dysfunction, especially in patients without epicardial stenosis. A Myocardial Perfusion Reserve (MPR) (ratio of MBF during maximal vasodilation to MBF at rest) < 2 indicates impaired coronary microcirculation, and it is associated with increased cardiovascular risk even in the absence of CAD. PET, although clinically valuable, is being underutilized due to high cost, limited availability, and radiation exposure. Emerging technological advances, including the development of high-sensitivity 3D PET scanners, aim to overcome such limitations with the reduction in radiation without compromising the quality of the assessment [8,9].

**Table 1:** Non-invasive imaging techniques to assess coronary microvascular dysfunction.

Imaging Modality	Methodology Protocol and Agents Used	Index Derived	Advantages	Disadvantages
PET	− Resting and stress imaging perfusion − Agent: radioisotopes, vasodilators	MPR < 2	− Gold standard technique − High accuracy − High reproducibility	− High cost − Does not exclude CAD − Time-consuming − Limited availability − Radiation exposure − Requires specialized tracers and equipment
CMR	− Resting and stress imaging perfusion − Agents: gadolinium-based contrast agents, vasodilators	MPRI < 2	− No radiation exposure − High spatial resolution	− Limited availability − Time-consuming − High cost − Contraindicated in some implants or claustrophobia − Does not exclude CAD
CT	− Resting and stress imaging perfusion − Agents: iodine-based contrast agents, vasodilators	MPR < 2	− Excellent anatomical assessment of coronary arteries	− Radiation exposure − Limited availability − High cost − Lacks validation
Doppler TTE	− Pulse wave on the LAD artery − Measurement of velocity at rest and stress − Agent: vasodilators (e.g., adenosine, dipyridamole)	CFR < 2	− No radiation exposure − Low cost − Widely available	− Operator-dependent − Poor acoustic window − Poor reproducibility − Does not exclude CAD − Limited to proximal coronary segments
MCE	− Assessment of Myocardial Blood Flow − Agent: Microbubble contrast agents	MBF < 2	− No radiation exposure − Low cost	− Does not exclude CAD − Lacks validation

CAD: Coronary Artery Disease, CFR: Coronary Flow Reserve, CMR: Cardiac Magnetic Resonance, CT: Cardiac Computer Tomography, LAD: Left Anterior Descending Artery, MBF: Myocardial Blood Flow, MCE: Myocardial Contrast Echocardiography, MPR: Myocardial Perfusion Reserve, MPRI: Myocardial Perfusion Reserve Index, PET: Cardiac Positron Emission Tomography, TTE: Transthoracic Echocardiography.

#### 2.2.2. Cardiac Magnetic Resonance (CMR)

CMR can also be used for the assessment of myocardial blood flow and CMD, using contrast agents (gadolinium-based) and stress vasodilators (i.e., adenosine or dipyridamole). In contrast to PET, CMR has no radiation exposure and has high spatial resolution. Impaired coronary microcirculation is indicated, measuring an MPR index < 2. The technique has several disadvantages, including high costs, dependence on renal failure, limited availability, and being extremely time-consuming [10,11]. Recent studies have proposed different cut-off values for the MPR index, depending on age and sex [12].

#### 2.2.3. Cardiac Computer Tomography (CT)

A combination of CT coronary angiography (CTCA) and cardiac CT perfusion (CTP) may be a valuable method for the assessment of macrovascular and microvascular disease during the same procedure. Vasodilators, as mentioned above, in combination with iodine-based contrast agents, are used for assessment of MBF (at rest and hyperemia). Disadvantages of the technique include a lack of validation with large studies and radiation exposure [13].

#### 2.2.4. Transthoracic Echocardiography (TTE) and Myocardial Contrast Echocardiography (MCE)

Transthoracic echocardiography is a reliable, inexpensive, and radiation-free technique for the evaluation of impaired coronary microcirculation. Using Doppler echocardiography, we can measure the maximal flow velocity at rest and after stress agent administration, often in the Left Anterior Descending (LAD) artery; their ratio is known as the coronary flow reserve (CFR). However, if visualization of the LAD artery is difficult, measurement can be obtained in other large coronary branches. The first-choice vasodilator agents used for maximal diastolic flow assessment at stress are adenosine (0.14 mg/kg/min for 2–4 min) and dipyridamole (0.84 mg/kg over 6 min). CFR (ratio of hyperemic to rest absolute myocardial flow) is a marker of CMD, and values < 2 identify impaired coronary microcirculation after exclusion of significant stenoses in epicardial arteries [14]. In addition to this, myocardial contrast echocardiography can be a useful technique for assessing coronary vasodilatory capacity, measuring myocardial blood flow (MBF) with a cut-off value of <2. However, the technique is not well spread in everyday clinical practice, mainly due to a lack of validation [15].

In the era of artificial intelligence (AI), automated programs have the potential to improve automation, standardization, and quantitative reproducibility in the measurement of CMD indices. In PET myocardial perfusion imaging, deep-learning models may automatically detect impaired myocardial flow reserve and predict patient risk directly from perfusion polar maps, thus enhancing diagnostic and prognostic yield [16]. For CMR perfusion, AI may enable automated quantitative segmentation and automatic calculation of myocardial perfusion reserve (MPR) [17]. Using deep-learning models, AI enhances estimation of the arterial input function, simplifying workflows and improving reproducibility across centers [18]. In CT perfusion, AI has been shown to automate myocardial segmentation and motion correction, reducing radiation dose, improving image quality, and shortening analysis time [19]. In Doppler transthoracic echocardiography, AI algorithms can track and trace the sample volume of pulsed-wave Doppler in the left anterior descending artery and automatically calculate CFR velocity both at baseline and during hyperemia. The development of models capable of identifying and analyzing coronary flow velocity patterns may substantially reduce intra- and inter-observer variability while also decreasing analysis time [20,21]. Finally, in MCE, AI-based methods can achieve myocardial segmentation with accuracy comparable to conventional techniques and, in parallel, substantially reduce analysis time and manual workload [22]. More broadly in echocardiography, AI can also perform automated quantification of established parameters such as left ventricular ejection fraction (LVEF), global longitudinal strain (GLS), and myocardial work (MW) related indices, further contributing to reproducibility and efficiency (Table 2).

**Figure 1:**
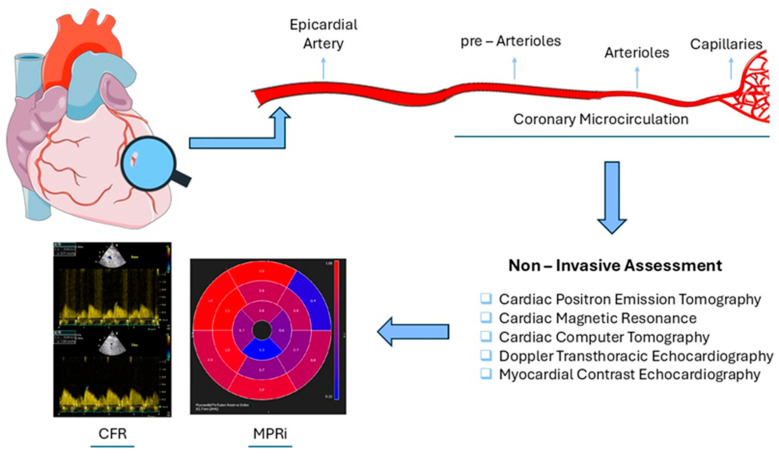
Impaired coronary microcirculation—non-invasive assessment. CFR: Coronary Flow Reserve, MPRi: Myocardial Perfusion Reserve index.

In summary, non-invasive techniques are generally safer than invasive ones, and some—such as PET—have already been validated. However, many non-invasive methods still require validation and large prospective studies to establish standardized cut-off values, as is the case with myocardial contrast echocardiography (MCE). On the other hand, invasive techniques offer the added advantage of directly assessing epicardial arteries. This contrasts with non-invasive methods, where only cardiac CT perfusion, particularly when used with CT coronary angiography (CTCA), can evaluate epicardial stenosis. Nevertheless, Doppler TTE and MCE have the benefit of no radiation exposure, while they are significantly less expensive than invasive procedures or other imaging modalities such as MRI or PET perfusion. (Figure 1).

## 3. Left Ventricular Systolic Function

**Figure 2:**
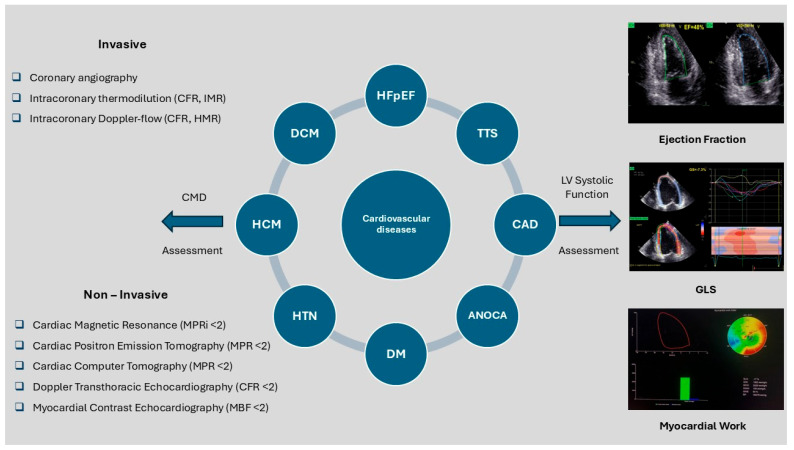
Assessment of CMD and LV systolic function across cardiovascular diseases. ANOCA: Angina With No Obstructive Coronary Artery Disease, CAD: Coronary Artery Disease, CFR: Coronary Flow Reserve, CMD: Coronary Microvascular Dysfunction, MBF: Myocardial Blood Flow, MPR: Myocardial Perfusion Reserve, MPRI: Myocardial Perfusion Reserve Index, DCM: Dilated Cardiomyopathy, DM (Diabetes Mellitus), GLS (Global Longitudinal Strain), HCM: Hypertrophic Cardiomyopathy, HTN: Hypertension, Hfpef: Heart Failure With Preserved Ejection Fraction, HMR: Hyperemic Microvascular Resistance, LV: Left Ventricular, MBF: Myocardial Blood Flow, MPR: Myocardial Perfusion Reserve, MPRI Myocardial Perfusion Reserve Index, TTS: Takotsubo Syndrome.

The assessment of left ventricular (LV) systolic function is crucial for the management of patients with cardiovascular diseases. (Figure 2) LVEF remains the gold standard systolic function echocardiography index. However, it has important physiological limitations (load dependency, presence of mitral regurgitation), technical limitations (dependence upon LV geometry, heart rate and rhythm, image quality, acoustic window, operator experience, large intra- and inter-operator variability), and limited sensitivity for detecting early LV dysfunction [23]. Speckle-tracking echocardiography (STE) has better sensitivity through measurement of GLS, a parameter that is considered extremely valuable for recognizing subclinical systolic dysfunction before LVEF begins to deteriorate. STE quantifies the motion of myocardial speckles during the cardiac cycle. Speckles are formed by the interaction of ultrasound waves and the myocardial tissue, and they can be tracked consistently in the longitudinal, circumferential, and radial axes from any imaging plane. Myocardial work (MW)-related indices combine myocardial strain with non-invasively estimated LV pressures for more in-depth analysis of cardiac function, estimating afterload, which is particularly important in clinical scenarios where using only LVEF or GLS as an index may not be sufficient. However, echocardiographic assessment of myocardial work still presents several limitations. High-quality two-dimensional imaging is necessary for its accuracy, and myocardial strain tracking is compromised by poor image quality, arrhythmias such as atrial fibrillation, and low frame rates. To guarantee a reliable estimation of afterload, blood pressure should be taken simultaneously and in the position at which the echocardiographic study is performed. Finally, the myocardial work analysis that is currently available is limited to a single vendor platform—commercial software— while standardized cut-offs are still lacking [24].

## 4. Coronary Microcirculation and Systolic Function in Cardiovascular Pathologies

### 4.1. Hypertension

Hypertension is an important risk factor for cardiovascular disease that significantly affects cardiac morbidity and mortality. It is associated with functional and structural changes in the myocardium and the arterial wall. Vascular endothelial dysfunction, median thickening of arterioles, and perivascular fibrosis lead to alterations in coronary microcirculation. Many studies have shown that coronary microcirculation, assessed by CFR, is impaired in hypertensive patients (from early stage to resistant hypertension) [25]. However, the impairment of CFR in these patients is independent of the presence and degree of LV hypertrophy.

Through the years, several groups have studied the correlation between CMD and LV systolic function in hypertension [26]. A study showed that impaired CFR contributed to exercise-induced myocardial ischemia and LV systolic dysfunction in hypertensive patients [27,28]. Caliskan et al. studied 170 patients with pre-hypertension and hypertension using Doppler TTE; patients were divided into an impaired CFR and a normal CFR group (using a cut-off value of CFR < 2.5). They reported that LV diastolic function parameters were more severely impaired in the CFR < 2.5 group, but there was no significant difference according to LV systolic function (assessed by LV ejection fraction) [29], indicating that other more sensitive indices should be used to assess for subclinical myocardial dysfunction in these patients.

The correlation between CFR (assessed by Doppler TTE and adenosine stress echocardiography) and LV systolic function (assessed by tissue Doppler imaging) was studied in 90 hypertensive patients (never treated) and 30 control subjects. Hypertensive patients were divided into CFR < 2.5 and CFR ≥ 2.5 subgroups. Patients with CFR < 2.5 were found to have a smaller increase in S’ after adenosine infusion, compared with the control and the CFR ≥ 2.5 group [30]. Five years later, the same research team, using the same procedure for the evaluation of CMD, investigated the relationship between impaired microcirculation and LV myocardial systolic function, using GLS, in 320 untreated hypertensive patients and 160 normal subjects. They showed that decreased CFR was strongly associated with impaired GLS [31]. Finally, Evangelou et al. studied 47 hypertensive and 20 healthy subjects, without known CAD, using TTE and dipyridamole stress echocardiography (DIPSE); CFR and GLS were measured at rest and after dipyridamole infusion. DIPSE-induced improvement in GLS was associated with higher CFR, only in hypertensive patients, indicating thus a strong pathophysiological association between coronary microcirculatory function and myocardial systolic function in these patients. These results, as well as their clinical significance, need to be validated in larger studies due to the limitations related to the small sample size [32].

### 4.2. Diabetes Mellitus (DM)

DM is a major cause of cardiovascular events by promoting inflammatory and atherosclerotic processes. DM leads to decreased nitric oxide bioavailability, endothelial dysfunction, and myocardial fibrosis, which in turn result in deregulation of coronary microcirculation and LV systolic and diastolic dysfunction.

Liu et al. studied the association of CMD with LV systolic function in 71 patients with type 2 DM (T2DM) and 30 healthy individuals who underwent CMR. The T2DM group was divided into newly diagnosed DM (<5 years) and long-term DM (>5 years), while CAD was excluded in all subjects using coronary CT angiography. CMR perfusion [upslope, time to maximum signal intensity, and max signal intensity] and LV deformation [global peak strain, peak systolic strain rate, and peak diastolic strain rate] indexes were measured and compared. The investigators reported that coronary microcirculation was impaired in the T2DM group and was associated with the duration of medical treatment. Additionally, they showed that both indices of myocardial perfusion (time to maximum signal intensity and upslope) were independently associated with peak longitudinal systolic strain rate, pointing to a possible mechanism linking subclinical LV systolic dysfunction and poor myocardial perfusion in T2DM patients [33]. Another pilot study investigated the possible association of CMD and LV systolic function in 32 patients with T2DM. All subjects underwent TTE and CMR with adenosine administration, and CFR was assessed by the ratio of coronary sinus flow at hyperemia to coronary sinus flow at rest. No correlation between CFR and LV systolic dysfunction (assessed by GLS) was reported in that study. However, this study was severely limited by the small sample size and the lack of a control group [34].

On the other hand, 45 patients with T2DM, a positive stress imaging test, and coronary angiogram without significant stenosis, and 35 healthy individuals were studied with TTE, strain imaging, DIPSE, and CFR measurement using Doppler echocardiography. The study showed that CFR and GLS were impaired in the T2DM group, and there was a significant association between these echocardiographic indices. Although the small sample size limited the ability to make definitive conclusions, the findings suggest that CMD in patients with DM and normal EF may contribute to reduced LV systolic function [35]. Finally, Ikonomidis I. et al. studied 40 first-degree relatives of T2DM patients with normal oral glucose tolerance test (OGTT), 40 dysglycemic, and 20 healthy subjects with normal OGTT. They reported that CFR (using Doppler echocardiography) was positively associated with LV systolic function (GLS) in first-degree relatives and dysglycemic subjects. However, the young study population, along with the limited sample size of the study, limits the potential extension of these findings to an older population [36].

### 4.3. Dilated Cardiomyopathy (DCM)

Patients with Dilated Cardiomyopathy (DCM) are at high risk of mortality. Many studies have shown that coronary microcirculation, evaluated by perfusion imaging techniques, is impaired in patients with DCM. In a large prospective study of patients with DCM, MPR and stress MBF were found to be lower compared to controls. MPR was also found to be associated with aerobic exercise capacity, an important prognostic marker of mortality in DCM patients [37]. Ma et al. studied 30 patients with DCM and 30 control individuals, using MCE, TTE, and two-dimensional speckle tracking echocardiography. MBF, GLS, and circumferential strain (GCS) were measured, and a correlation between LV microcirculation perfusion and GCS was reported [38]. In addition, another study based on CMR indexes demonstrated that microvascular dysfunction may contribute to impaired LV systolic function in patients with idiopathic DCM [39]. Furthermore, MBF and MPR (estimated by PET) have been found to be more severely impaired in patients with the dilated phase of hypertrophic cardiomyopathy (HCM) compared to patients with DCM, leading to increased cardiovascular events and worse prognosis [40].

### 4.4. Hypertrophic Cardiomyopathy (HCM)

Various studies have shown the association of CMD with LV systolic function in HCM patients. Using CMR perfusion imaging, a study showed that CMD was present in patients with HCM before LV hypertrophy was established. Stress MBF was lower than MBF at rest in these patients, leading to myocardial ischemia, fibrosis, and subclinical LV systolic dysfunction [41]. Olivotto et al. studied 51 patients with HCM, in whom coronary microcirculation was assessed by PET using dipyridamole. In these patients, severe CMD was found in association with LV systolic dysfunction and LV remodeling [42]. In another study by Garcia Brás et al., 75 patients with HCM and no known history of CAD were divided into obstructive and non-obstructive HCM groups. Stress perfusion CMR, 2-dimensional speckle tracking echocardiography, and assessment of Myocardial Work indices were performed in all patients. In this elegant study using novel tools, a correlation between perfusion defects and myocardial work parameters was reported, irrespective of the presence of fibrosis, LV hypertrophy, or obstruction [43].

### 4.5. Takotsubo Syndrome (TTS)

TTS, also known as stress-induced myocardiopathy, is characterized by impairment of left and/or right ventricular systolic function, electrocardiographic ST-segment abnormalities, increased levels of troponin, and absence of significant stenosis on epicardial arteries. Most patients present with LV wall motion abnormalities in the mid-apical regions with hyperkinesis of the basal segments, referring to a recent stressful event [44,45]. Studies have observed that coronary microcirculation is impaired in patients presenting with TTS, while its function is shown to be recovering in parallel with the improvement of LV wall abnormalities [46]. Meimoun et al. studied 20 patients with TTS who underwent Adenosine Stress Echocardiography with CFR evaluation and wall motion score measurement. CFR was found to be significantly correlated to wall motion score, revealing thus a close correlation of CMD and LV systolic function parameters in the acute phase and indicating that CMD may play a key role in the pathogenesis of TTS. CFR was not measured immediately upon patient admission but within 48 h, when patients were in stable condition, probably suggesting that it could have been even worse if measured earlier [47].

### 4.6. Obstructive CAD

Obstructive CAD and CMD may cause myocardial ischemia in epicardial arteries and microcirculation and lead to impairment of LV systolic function [48]. Coronary microcirculation may be impaired, even after successful percutaneous coronary intervention, due to plaque embolism, neoatherosclerosis, and vasomotor abnormalities [49]. The CIRT -CFR trial recruited 50 patients who had a previous myocardial infarction or multivessel CAD and either T2DM or metabolic syndrome. All participants performed PET/CT using dipyridamole or adenosine, and CFR was calculated as the ratio of MBF at hyperemia to MBF at rest. At the time PET was performed, GLS as a marker of LV systolic function and other indices of LV diastolic dysfunction were assessed. This prospective study showed that impaired CFR (cut-off value < 2) correlated with myocardial strain, and this correlation was independent of metabolic markers [50]. Logstrup et al. had already reported this association in a large study with 180 participants who had recently suffered from an acute coronary syndrome, using Doppler echocardiography to assess CFR, GLS, and regional longitudinal systolic strain [51]. Another study based on MCE for evaluation of CMD and Myocardial Work parameters for LV systolic function highlighted the association between microvascular perfusion and Global Work Index in patients with ST-elevation myocardial infarction treated with primary percutaneous coronary intervention, indicating that Myocardial Work may be used for detection of CMD and early deterioration of LV systolic function [52].

### 4.7. Angina with No Obstructive Coronary Artery Disease (ANOCA)

ANOCA (angina with no obstructive coronary artery disease) is a syndrome characterized by angina that is not accompanied by significant stenoses in epicardial arteries. Many studies have demonstrated that patients with ANOCA have impaired microcirculation (Table 3). CMD in ANOCA patients has been reported to increase the risk of adverse cardiovascular events and mortality [53].

Liu et al. and Jovanovic et al. studied the relationship between CMD and LV systolic function, enrolling 78 and 67 patients with ANOCA, respectively. All patients underwent adenosine stress echocardiography, and CFR was measured using Doppler echocardiography. CMD was associated with LV systolic function (assessed by GLS) both at rest and during exercise [54,55]. Xing et al. revealed similar results, using the same methods, comparing ANOCA patients to patients with classical CAD and control subjects [56]. Using dipyridamole as a vasodilator agent in studying coronary microcirculation via Doppler echocardiography, GLS at rest, GLS after dipyridamole stress echocardiography, and GLS reserve were found to be significantly lower in patients with ANOCA and CMD, thus reflecting subclinical LV systolic dysfunction in these patients [57,58]. Li et al. found that, although GLS was not significantly different between CMD and non-CMD groups in ANOCA patients, Myocardial Work, and especially the indices Global Work Index and Global Constructive Work, were found to be associated with CFR, while Global Constructive Work was associated with CMD [59]. Finally, Liu et al. showed that Myocardial Work could be a valuable marker for ANOCA with CMD diagnosis. Furthermore, they also showed that increased myocardial waste work in CMD subgroups at stress may be caused by increased cardiac asynchrony [60].

### 4.8. Heart Failure with Preserved Ejection Fraction (HFpEF)

Heart failure (HF) is a complex clinical syndrome that presents with symptoms such as shortness of breath, fatigue, and bilateral ankle edema. The main causes of HF are CAD, arterial hypertension, valvular heart disease, and cardiomyopathies. Diagnosis of HF with Preserved Ejection Fraction (HFpEF) is based on symptoms and clinical findings of HF, LV ejection fraction > 50% and findings of structural and/or functional cardiac abnormalities with the presence of LV diastolic dysfunction/ elevated LV filling pressures, combined with increased Natriuretic Peptide (NP) levels. The main echocardiographic findings indicating the presence of LV diastolic dysfunction/elevated LV filling pressures are: LV hypertrophy (LV mass index > 95 g/m^2^ in females or >115 g/m^2^ in males), left atrial enlargement (LA volume index > 34 mL/m^2^ for patients in sinus rhythm and >40 mL/m^2^ for patients in atrial fibrillation), the ratio of early E/e’ > 9 at rest, pulmonary arterial systolic pressure > 35 mmHg and tricuspid regurgitation velocity max >2.8 m/s [61].

HFpEF often co-exists with systemic comorbidities, which lead to impairment of coronary microcirculation through an inflammatory pathway. CMD, as indicated by reduced CFR, is believed to play an important diagnostic and prognostic role in these patients. In patients with HFpEF and moderate or severe aortic stenosis, CFR has been reported to be a predictor of mortality [62]. According to Paulus and Tschope, several comorbidities in patients with HFpEF (e.g., hypertension, DM, chronic kidney disease) lead to an inflammatory process, which in turn results in deregulation of coronary microcirculation and LV wall stiffness [63]. Systemic inflammation causes a reduction in NO bio-availability, leading to a reduction in titins-connectins and finally to LV wall thickening. Additionally, this inflammatory response decreases metalloproteinases, leading to further LV stiffness. Abnormalities in coronary microcirculation are correlated with endothelial dysfunction, microvascular rarefaction, and inflammation. Endothelial dysfunction causes conversion of endothelial cells into fibroblasts, leading to myocardial dysfunction and fibrosis. Myocardial stiffness and fibrosis impair diastolic and systolic mechanics and lead to myocardial dysfunction [64,65]. Among other comorbidities, CMD in chronic kidney disease is frequently accompanied by LV diastolic impairment, whereas LV systolic deformation may remain preserved in earlier stages [66,67].

Many studies have shown the association of CMD with indices of LV systolic function in patients with HFpEF. Using invasive methods, CFR was found to be decreased in patients with HFpEF and CMD and correlated with LV dysfunction in these patients [68,69]. Kato et al. evaluated LV systolic dysfunction and CMD in 163 patients with HFpEF using CMR; MBF was measured as coronary sinus blood flow/LV mass, and CMD was evaluated by CFR, as the ratio of MBF after adenosine triphosphate administration to MBF at rest. Patients were divided into two groups based on CFR (cut-off value < 2), and a significant correlation was reported between CFR and GLS, CFR and GCS, as well as CFR and RV longitudinal strain. During follow-up, CFR was found to be more severely impaired in patients with adverse events [70], indicating that CFR assessed by Cine CMR could be a valuable prognostic tool for patients with HFpEF. In a multicenter observational study (PROMIS-HFpEF), 202 patients with HFpEF and no known history of CAD were studied using adenosine stress Doppler TTE and measurement of CFR. Of those, 151 patients had CMD (CFR < 2.5), and impaired microcirculation was found to be associated with subendocardial myocardial dysfunction. In patients with HFpEF and CMD, lower CFR was associated with worse LV GLS and RV free wall myocardial strain [71]. In sophisticated studies using indices of Myocardial Work, it was shown that Global Work Efficiency, Global Work Index, and Global Constructive Work were significantly reduced in HFpEF patients, implying that further research into the correlation between CFR and Myocardial Work parameters would be of great value [72,73] (Table 4).

## 5. Conclusions

Myocardial systolic function has been assessed non-invasively for many decades using various echocardiographic indices. In more recent years, GLS and Myocardial Work–related indices have been developed, allowing the detection of subclinical myocardial ischemia and early deterioration of LV systolic function, especially in patients with normal ejection fraction. Currently, coronary microcirculation can also be assessed non-invasively using a variety of methods such as PET, CMR, CT perfusion, Doppler TTE, and MCE, each with its own advantages and disadvantages. CFR measurement with Doppler TTE, a low-cost method with wide availability and no radiation exposure, is well correlated with PET [74], which is considered the gold standard technique.

Coronary microcirculation is known to be impaired in many cardiovascular diseases, and CMD might be associated with poor clinical outcomes in these patients. Several studies have also investigated the correlation between CMD and myocardial systolic dysfunction in various cardiovascular pathologies, indicating this association as the potential link of CMD to poor prognosis. The association of CMD and myocardial systolic dysfunction highlights the pathway of coronary microcirculation impairment as a possible target for therapeutic strategies, while it indicates the potential role of CMD indices as valuable prognostic markers in various cardiovascular diseases. Thus, routine assessment of coronary microcirculation may be a valuable addition to risk stratification strategies in patients with cardiovascular diseases, such as HFpEF and ANOCA. The standardization of non-invasive imaging techniques for CMD assessment and their testing in prospective multicenter trials is needed to establish the diagnostic and prognostic value of coronary microcirculation-specific indices and to assess whether CMD-guided management may impact clinical outcomes.

## Figures and Tables

**Table 2:** AI-enabled non-invasive CMD assessment.

Imaging Modality	AI Tasks	Advantages
PET	− Automatic MPR calculation	− Short analysis time
CMR	− Automated myocardial segmentation − Automatic MPR calculation − AIF correction	− Improved reproducibility − Simplified workflows − Short analysis time
CT	− Automated myocardial segmentation − Motion correction	− Reduced radiation dose, − Improved image quality − Short analysis time
Doppler TTE	− Auto-tracking/ tracing of PW sample volume − Automatic CFR calculation	− Reduced intra-/inter-observer variability − Short analysis time
MCE	− Automated myocardial segmentation	− Short analysis time − Reduced manual workload

AI: Artificial Intelligence, AIF: Arterial Input Function, CFR: Coronary Flow Reserve, CMD: Coronary Microvascular Dysfunction, CMR: Cardiac Magnetic Resonance, CT: Cardiac Computer Tomography, MCE: Myocardial Contrast Echocardiography, MPR: Myocardial Perfusion Reserve, PET: Cardiac Positron Emission Tomography, PW: Pulse Wave, TTE: Transthoracic Echocardiography.

**Table 3:** Studies showing a positive correlation between indices of coronary microvascular dysfunction and indices of myocardial systolic dysfunction in patients with angina with no obstructive coronary artery disease (ANOCA).

Study	ANOCA Patients (N)	Technique	Agent	CMD Index	Systolic Function Index
**Liu et al.,** **2021**	78	Doppler TTE	Adenosine	CFR < 2	GLS
**Xing et al.,** **2020**	67	Doppler TTE	Adenosine	CFR < 2.5	GLS
**Jovanovic et al.,** **2020**	70	Doppler TTE	Adenosine	CFR < 2	GLS
**Michelsen et al.,** **2018**	963	Doppler TTE	Dipyridamole	CFR < 2	GLS
**Tagliamonte et al.,** **2023**	59	Doppler TTE	Dipyridamole	CFR < 2	GLS
**Li et al.,** **2023**	97	Doppler TTE	Adenosine	CFR < 2	MW
**Liu et al.,** **2023**	78	Doppler TTE	Adenosine	CFR < 2.5	MW

TTE: Transthoracic Echocardiography, CFR: Coronary Flow Reserve, GLS: Global Longitudinal Strain, MW: Myocardial Work. N: number of patients.

**Table 4:** Studies show a positive correlation between CMD and LV systolic dysfunction across cardiovascular diseases.

Study	Patients (N)	CVD	Technique	Level of Evidence *
**Kozàkovà et al.** **(2003)**	60	HTN	Doppler TEE	Low
**Ikonomidis et al.** **(2008)**	100	HTN	Doppler TTE	Low
**Ikonomidis et al.** **(2012)**	90	HTN	Doppler TTE	Low
**Ikonomidis et al.** **(2018)**	320	HTN	Doppler TTE	Low
**Evangelou et al.** **(2020)**	47	HTN	Doppler TTE	Low
**Ikonomidis et al.** **(2022)**	80	DM	Doppler TTE	Low
**Liu et al.** **(2018)**	78	DM	CMR	Low
**D’ Andrea et al.** **(2010)**	45	DM	Doppler TTE	Low
**M. Takafuji et al.** **(2023)**	26	DCM	CMR	Low
**Olivotto et al.** **(2006)**	51	HCM	PET	Low
**Bras et al.** **(2023)**	75	HCM	CMR	Moderate
**Meimoun et al.** **(2009)**	20	TTS	Doppler TTE	Low
**Tanqueri et al.** **(2023)**	50	CAD	PET	Moderate
**Løgstrup et al.** **(2012)**	183	CAD	Doppler TTE	Low
**Jin et al.** **(2022)**	160	CAD	MCE	Low
**Liu et al.,** **2021**	78	ANOCA	Doppler TTE	Moderate
**Xing et al.,** **2020**	67	ANOCA	Doppler TTE	Low
**Jovanovic et al.,** **2020**	70	ANOCA	Doppler TTE	Low
**Michelsen et al.,** **2018**	963	ANOCA	Doppler TTE	Moderate
**Tagliamonte et al.,** **2023**	59	ANOCA	Doppler TTE	Low
**Li et al.,** **2023**	97	ANOCA	Doppler TTE	Moderate
**Liu et al.,** **2023**	78	ANOCA	Doppler TTE	Moderate
**Kato et al.,** **2021**	163	HFpEF	CMR	Moderate
**Shah et al.,** **2018**	202	HFpEF	Doppler TTE	Moderate

* Findings are reported according to GRADE certainty levels. ANOCA: Angina With No Obstructive Coronary Artery Disease, CAD: Coronary Artery Disease, CMD: Coronary Microvascular Dysfunction, CMR: Cardiac Magnetic Resonance, DCM: Dilated Cardiomyopathy, DM: Diabetes Mellitus, HCM: Hypertrophic Cardiomyopathy, HTN: Hypertension, Hfpef: Heart Failure With Preserved Ejection Fraction, LV: Left Ventricular, MCE: Myocardial Contrast Echocardiography, PET: Cardiac Positron Emission Tomography, TEE: Transesophageal Echocardiography, TTE: Transthoracic Echocardiography, TTS: Takotsubo Syndrome.

## Data Availability

Data is contained within the article.

## Abbreviations

2D-STE (Two-dimensional speckle tracking echocardiography), (ADSE (Adenosine stress echocardiography), ANOCA (Angina with No Obstructive Coronary Artery disease), AI (Artificial Intelligence), AIF (Arterial Input Function),BH_4_ (Tetrahydrobiopterin), CAD (Coronary artery disease), CBF (Coronary blood flow), CFR (Coronary Flow Reserve), CMD (Coronary microvascular dysfunction), CMR (Cardiac magnetic resonance), COX (Cyclooxygenases), CT (Computed tomography), CTCA (CT coronary angiography), CTP (CT perfusion), DCM (Dilated Cardiomyopathy), DIPSE (Dipyridamole stress echocardiography), DM (Diabetes Mellitus), FFR (Fractional flow reserve), GCW (Global Constructive Work), GLS (Global longitudinal strain), GWE (Global Work Efficiency), GWI (Global Work Index), HCM (Hypertrophic cardiomyopathy), HF (Heart failure), HTN (Hypertension), HFpEF (Heart Failure with Preserved Ejection Fraction), HMR (Hyperemic Microvascular Resistance), LAD (Left Anterior Descending), LV (Left ventricular), LVEF (Left ventricular ejection fraction), LVH (LV hypertrophy), MBF (Myocardial blood flow), MCE (Myocardial contrast echocardiography), MPR (Myocardial Perfusion Reserve), MPRI (Myocardial Perfusion Reserve Index), NO (Nitric oxide), Nox (NADPH oxidase), NP (Natriuretic Peptide), OGTT (Oral glucose tolerance test), PET (Positron emission tomography), PW pulse wave, ROS (Reactive oxygen species), RV (Right ventricular), STE (Speckle-tracking echocardiography), T2DM (Type 2 DM), TEE (Transesophageal Echocardiography), TTE (Transthoracic Echocardiography), TTS (Takotsubo Syndrome).
